# Beyond the List: Bioagent-Agnostic Signatures Could Enable a More Flexible and Resilient Biodefense Posture Than an Approach Based on Priority Agent Lists Alone

**DOI:** 10.3390/pathogens10111497

**Published:** 2021-11-17

**Authors:** Owen P. Leiser, Errett C. Hobbs, Amy C. Sims, George W. Korch, Karen L. Taylor

**Affiliations:** 1 Pacific Northwest National Laboratory, Seattle, WA 98109, USA; owen.leiser@pnnl.gov (O.P.L.); errett.hobbs@pnnl.gov (E.C.H.); 2 Pacific Northwest National Laboratory, Richland, WA 99354, USA; amy.sims@pnnl.gov; 3 Battelle National Biodefense Institute, LLC, Fort Detrick, MD 21072, USA; george.korch@st.dhs.gov

**Keywords:** bioagent-agnostic, biodefense, interactome, biosurveillance, select agents, countermeasures, host response, multi-omics, omics, biological signatures

## Abstract

As of 2021, the biothreat policy and research communities organize their efforts around lists of priority agents, which elides consideration of novel pathogens and biotoxins. For example, the Select Agents and Toxins list is composed of agents that historic biological warfare programs had weaponized or that have previously caused great harm during natural outbreaks. Similarly, lists of priority agents promulgated by the World Health Organization and the National Institute of Allergy and Infectious Diseases are composed of previously known pathogens and biotoxins. To fill this gap, we argue that the research/scientific and biodefense/biosecurity communities should categorize agents based on how they impact their hosts to augment current list-based paradigms. Specifically, we propose integrating the results of multi-omics studies to identify bioagent-agnostic signatures (BASs) of disease—namely, patterns of biomarkers that accurately and reproducibly predict the impacts of infection or intoxication without prior knowledge of the causative agent. Here, we highlight three pathways that investigators might exploit as sources of signals to construct BASs and their applicability to this framework. The research community will need to forge robust interdisciplinary teams to surmount substantial experimental, technical, and data analytic challenges that stand in the way of our long-term vision. However, if successful, our functionality-based BAS model could present a means to more effectively surveil for and treat known and novel agents alike.

## 1. Introduction

In the United States, biodefense and biosecurity policy as well as biological threat research largely center around several lists of priority agents including the Federal Select Agent Program (FSAP) Select Agents and Toxins list [1] which is intended to catalog and define security measures for those agents that pose an especially high risk to human, animal, and plant health, the National Institute of Allergy and Infectious Diseases (NIAID) Emerging Infectious Diseases/Pathogens list (catalogs both biodefense risks and emerging pathogens) [2], and the World Health Organization priority pathogens list [3] (highlights drug-resistant pathogens for which there is an urgent need to develop new antimicrobial therapies). List-based approaches such as these focus resources on detecting, characterizing, and countering agents that were part of nation-state bioweapons research programs or that have proven to be especially dangerous natural threats. Crucially, none of these frameworks prepare us to address mechanisms or threats posed by truly novel pathogens that by definition have not previously appeared on any list.

We argue that the biodefense and emerging infectious diseases communities should adopt a new strategy to augment—but not replace—list-based approaches. Others in the biodefense community have recognized how too heavily relying on lists limits our biothreat detection, diagnostic, and countermeasure capabilities; the 2018 National Academies of Sciences report on Biodefense in the Age of Synthetic Biology [4] states “an overreliance on the Select Agent List is a systematic weakness affecting many aspects of the United States’ current biodefense mitigation capability.” Specifically, we argue that the focus should expand to include characterizing host responses that are common to groups of pathogens.

The outcomes of this research would lay the foundation for identifying bioagent-agnostic signatures (BASs) of disease; we defined a BAS as a clinically measurable suite of biomarkers that accurately and reproducibly predicts the impacts of infection without a priori knowledge of an agent. Bioagent-agnostic signatures would incorporate various forms of data (e.g., information from multi-omic and single modality ‘omic studies, such as proteomic, metabolomic, lipidomic, transcriptomic, and genomic data) that describe host characteristics and responses or broadly identify the presence of a class of pathogen (e.g., lipopolysaccharide indicates a Gram-negative bacterium is present). These signatures would comprise multiple “readout” molecules such as RNAs, proteins, and immune metabolites. For example, the ‘omics analysis of peripheral blood mononuclear cells and plasma from Ebola virus-infected patients identified subsets of host responses that correlated with either survival or severe disease outcomes [5,6].

We believe that this strategy shift could help unify existing research efforts; it could also serve as a basis for host-focused biosurveillance to complement pathogen-focused biosurveillance programs and determining which countermeasures to deploy or develop against a disease. Employing a BAS-based approach to biodefense would shift the community’s focus from characterizing specific pathogenic taxa to a framework that hinges on identifying functional interactions in host networks which drive or enable pathogenesis; recentering our biodefense posture in this way could make it easier to control future biological threats. These signatures would (1) be demonstrative of the specific interactions a pathogen employs to usurp host processes and (2) represent evolutionarily conserved host-response patterns to a wide range of pathogens and toxins that elicit disease.

Because this approach would push the community to delineate broader and more consistent themes in host response to identify and mitigate disease, it might also help dampen rapid priority swings as new threats emerge (e.g., SARS-CoV-2 in 2019, the Amerithrax attacks of 2001) [7,8]. Although we believe that the biodefense community should incorporate a BAS-based approach, we acknowledge that it will likely never completely supplant the established list-based (known pathogen) paradigm. For example, regulatory authorities and funding agencies will still need to clearly articulate which pathogens researchers should prioritize. Additionally, research on therapies directed against pathogens and toxins themselves (i.e., antibiotic, antiviral, antifungal, antiparasitic, and antitoxin drugs) must continue.

We recognize that our proposal constitutes a major shift in the U.S. biodefense posture and will likely take many years to realize. Biologists must make fundamental advances in experimental design, technology, and data analysis to identify BASs that robustly classify similar courses of disease elicited by disparate pathogens and toxins. With BASs in hand, translational scientists and engineers will need to develop assays and procedures to operationalize them to protect human health and to conduct biosurveillance. Some BASs may only describe correlations to disease; scientists will likely need to understand the causal factors driving pathogenesis to fully leverage BASs as tools for countermeasure deployment and development and to triage cases based on predicted disease severity.

We will briefly outline representative research with the potential to identify signals that scientists could incorporate into BASs and current impediments to implementing this approach. We will focus on humans as the host of interest, but this concept could be equally valid in categorizing animal and plant host responses.

## 2. Common Themes in Host Response Could Form the Basis for Establishing BASs

**Figure 1:**
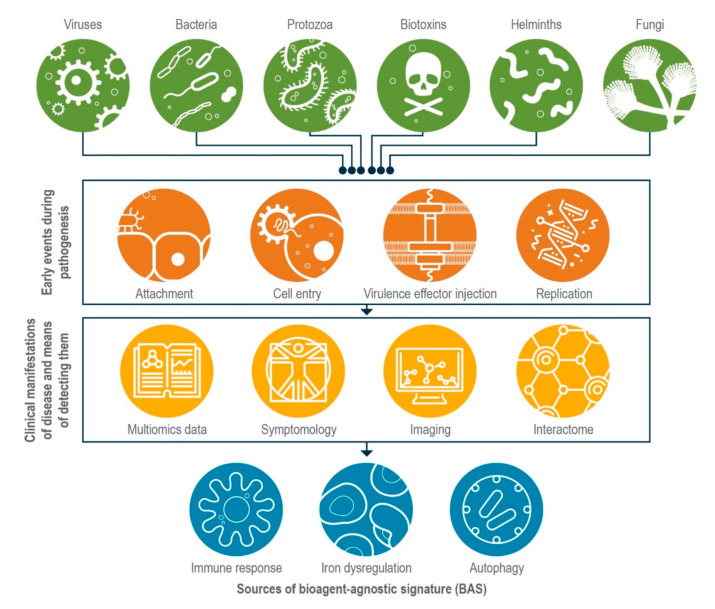
Bioagent-agnostic signatures (BASs) would reflect perturbations to host processes. Early events in pathogenesis or intoxication from different agents (green circles) are thematically similar; for example, a pathogen or toxin needs to attach and enter a target cell to initiate an infection or intoxication (orange circles). Scientists can employ a variety of tools to interrogate host responses to these insults (yellow circles) and identify signals in host processes that they could integrate to form BASs (blue circles). In addition to examples discussed in the text, biologists also could mine host responses to infection by protozoa [15], helminths [16], and fungi [17] as well as toxin exposure [18,19,20].

Host–pathogen studies generally revolve around individual host–pathogen interactions (e.g., *Bacillus anthracis* infection in humans); however, commonalities exist in host responses to disparate pathogens, which could serve to nucleate the development of BASs (Figure 1). Adding to an already large body of knowledge on host–pathogen interactions [9,10,11], ’omics approaches are beginning to illuminate the ways that host and pathogen pathways work in concert to give rise to disease phenotypes [12,13,14]. Below, we briefly describe three broad types of host responses that scientists could measure and incorporate into BASs to enable response pattern recognition and biosurveillance; we also suggest further study of host–pathogen interactomes as a way of identifying complex, multi-omic BASs.

### 2.1. Host Innate Immune Response during Infection

The innate immune response is evolutionarily ancient, serves as the first line of host defense against infection in humans, and is a potentially rich source of signals to incorporate into BASs [21]. Cells in the innate immune system harbor pattern-recognition receptors that recognize and are strongly stimulated by specific pathogen-associated molecular patterns (PAMPs). Upon PAMP activation, the innate immune system triggers signaling cascades that gird other cells against infection and recruit adaptive immune cells to help clear pathogens [22,23,24,25]. The innate immune system integrates signals from different PAMPs, which shapes the nature of its response [26]. Immune activation is complex and finely tuned to the type, replication status, virulence, and viability of an infectious agent as well as the route of infection [27].

We speculate that scientists could further parse these interactions to establish host–pathogen pattern typologies that are indicative of bacterial infection. For example, antigen-presenting cells produce interleukin 12 (IL-12) in response to stimuli such as lipopolysaccharide, protozoal extract, and viral infection, which promotes a Th1 adaptive response to clear intracellular pathogens, including bacteria, protozoa, and viruses [28,29,30,31,32,33,34,35]. Additionally, bacteria often perturb and manipulate kinase signaling pathways such as NF-κB [36] and MAPK [37,38,39] to establish an infection niche and promote intracellular survival.

Similar opportunities could exist for viral pathogens. Hosts activate interferon signaling pathways in response to viral infection, which many viruses in turn manipulate as part of their pathogeneses and makes these pathways attractive as BAS inputs. Multiple cell types can sense viral PAMPs and subsequently activate the innate immune system to produce interferons and other cytokines [22,40,41,42,43,44]. Ultimately, these pathways work together to (1) induce an antiviral state that restricts viral replication in both infected and neighboring cells and (2) shape the nature of the adaptive immune response [45,46,47].

Viruses also reprogram innate immune response networks to establish productive infections. For example, herpesviruses encode proteins that inhibit different forms of programmed cell death [48,49,50]. Using a combined transcriptomics and proteomics approach, Menachery et al. [51] identified overlapping and unique mechanisms by which the taxonomically unrelated H5N1 influenza virus and MERS-CoV modify host histone methylation states in human airway epithelial cells to directly repress interferon cascades. In contrast, SARS-CoV-1 traps key innate immune response transcription factors in the cytoplasm to prevent interferon-stimulated gene expression [52].

### 2.2. Dysregulation of Iron Homeostasis during Infection

Iron homeostasis underpins many processes in both healthy and diseased host cells as well as pathogens, which suggests that associated pathways could be a rich source of information for creating BASs. Hosts closely control iron levels, which protects them against both infection [53] and reactive oxygen species that can damage host cells and that readily form when free iron donates electrons to oxygen [54]. Hosts effect control by producing iron-chelating proteins to scavenge iron from blood and other tissues [55,56] and employing transferrins to selectively import iron into host cells [57].

Iron acquisition is a key virulence mechanism in both Gram-positive and Gram-negative bacteria that hosts actively resist. Bacterial pathogens express iron-scavenging systems (e.g., siderophores [58,59], hemophores [60,61], and specific uptake systems for each) under low iron conditions that compete with host-scavenging systems [62]. In response to infection, neutrophils produce the siderophore-binding molecule siderocalin, which prevents the bacterial uptake of iron-siderophore complexes [63], and macrophages produce ferritin to sequester free intracellular iron [64]. Additionally, the dual regulatory hormone/antimicrobial peptide hepcidin acts on macrophages during bacterial infection to downregulate the only known iron exporter in humans, ferroportin, which additionally sequesters iron from pathogens [65].

Dysregulation of iron homeostasis is also a key factor in some viral pathogeneses; in some cases, cells with disrupted iron homeostasis are more susceptible to infection and transferrin receptors can serve as cellular receptors for viral entry [66]. In the past year, research has demonstrated COVID-19 patients with markers of dysregulated iron homeostasis, including both anemia and ferritinemia, experienced worse disease outcomes than patients with normal iron levels [67,68,69,70]. Additionally, several other human viruses, including respiratory syncytial virus, hepatitis C, and human immunodeficiency virus (HIV) [71,72,73] alter iron homeostasis during infection, which in some cases, clinicians have linked to increased severity of infection and poor patient outcomes [74,75].

### 2.3. Autophagy

Autophagy is the process by which host cells produce membrane vesicles to encapsulate intracellular materials and debris that lysosomes degrade and recycle into constituent components. Cells also employ autophagic pathways as a defense mechanism to engulf and destroy invading pathogens, in a process called xenophagy. Accordingly, pathogenic microorganisms have developed numerous mechanisms to manipulate or suppress autophagy upon host cell entry [76].

Bacteria such as *Salmonella typhimurium*, *Shigella flexneri*, and *Legionella pneumophila* block the recruitment of autophagic proteins [77,78] or escape phagosomes entirely [79] to avoid autophagy and establish an intracellular niche. *S. typhimurium* recruits a host protein (focal-adhesion kinase) to suppress autophagy in a way that also suppresses the interferon response [80]. In contrast, intracellular *Staphylococcus aureus* induces host autophagy as a mechanism to scavenge nutrients during infection, through extensive remodeling of central carbon metabolism [81]. In this case, researchers observed increased levels of phosphorylated host adenosine monophosphate-activated protein kinase (MAPK) and extracellular signal-related kinase (ERK), suggesting another potential signature indicative of infection.

Viruses also exploit autophagy in a complex manner during infection; host cell autophagy can exhibit both pro-viral and antiviral roles during infection [82]. Viruses balance induction of autophagy and apoptosis to maximize viral particle production and can induce incomplete autophagy to disrupt the formation or maturation of phagosomes [83,84,85], evade proteolytic degradation within phagosomes [86], and enhance replication [87,88]. Coronaviruses can both disrupt the formation and maturation of phagosomes as well as induce the formation of double-membraned vesicles from the endoplasmic reticulum of infected cells [89]. Dengue viruses exploit autophagic pathways to promote host lipid degradation as an energy source to enhance replication [90,91]. Many types of viruses disrupt and manipulate autophagic pathways; understanding the similarities and differences between these mechanisms could form the basis for additional BASs.

### 2.4. Exploiting the Interactome

Systems biology approaches can define “interactomes” of interconnecting proteins and molecules between host cells and pathogens; we anticipate higher-order analyses that compare interactomes across pathogeneses induced by different agents will reveal similarities that scientists could exploit in BASs [92,93]. For example, interaction networks highlight the multifaceted role epidermal growth factor receptor (EGFR) plays in pathogenicity; it acts as a key regulator during influenza virus infection [94] and is exploited by vaccinia virus to promote cell motility and the spread of infection in its host [95]. Host/pathogen interaction networks have also been defined in different organs during *Streptococcus pneumoniae* infection [96]. Griesenauer et al. [97] demonstrated the utility of an ‘omics approach (in this case, RNA-seq and metabolomics), for the first time investigating the interaction network between *Haemophilus ducreyi* and human hosts and characterizing the transcriptional response in both organisms. Studies such as these demonstrate the utility of large-scale computational methods to integrate ‘omic data to dissect complex host/pathogen interaction networks.

## 3. As They Mature, Multi-Omics Approaches May Reveal More BASs

Individual ‘omics technologies may suffice to generate some BASs; however, we expect that as multi-omics technologies develop, they will provide an increased resolution of different disease states. Individual ‘omics technologies (e.g., genomics, transcriptomics, proteomics, metabolomics) only measure events in a single type of analyte (e.g., DNA, RNA, protein, metabolite), which might not be fully indicative of the state of the larger biological system. In contrast, multi-omics technologies promise to connect events that transpire among different analyte layers [98] and better describe the current state of the system to predict subsequent events. Multi-omics technologies also connect information that more directly represent the genetic and environmental factors which influence a biological system.

As of 2021, multi-omics approaches are relatively immature; consequently, elucidating BASs will be experimentally and computationally challenging. Although scientists are actively researching advances in throughput and multimodal integration [99,100,101,102], some individual ‘omic—and by extension, their derivative multi-omic—technologies, are limited in the number of unique analyte species they can interrogate in one run [103,104,105,106]. Genetic and environmental factors as well as procedural details in execution that differ between experiments can influence results in ways that are difficult for scientists to ascertain; better approaches are required to ensure data are reproducible, and this should be a priority for research funding. Otherwise, scientists will not be able to ascertain BASs in broad populations, which would render BASs useless as the basis for a biodefense strategy. Scientists also need better models to eliminate nuisance technical variation that arises between different runs of the same single-modality ‘omics experiment (i.e., batch effects) [107,108]. Assuming scientists can address these problems, the large volume, missingness, and high dimensionality of multi-omics data force researchers to make hard choices about how to extract and analyze pertinent information; these choices will influence their ability to uncover relevant BASs.

## 4. Conclusions

Scientists will need to overcome substantial experimental, technical, and data-analytic challenges to identify robust BASs and implement them in practice. These hurdles will take time and focused effort to resolve. However, the research community is beginning to take steps in this direction. For example, the US Food and Drug Administration (FDA) recently granted 501(k) approval for a diagnostic that measures host responses to distinguish between bacterial and viral infections [109]. The toxicology community is also considering how to exploit multi-omics data for risk assessments [98]. If successful, our BAS framework presents a path to addressing threats posed by both known and novel pathogens. It could also provide a principled way to augment formal risk assessments for novel biothreats conducted under the ISO 35001:2019 standard [110].

Using BASs to define disease states and provide an early warning system for infection and outbreaks represents an important addition to the current list-based biosurveillance and biodetection model. In contrast to list-based models of biosurveillance, the BAS model focuses on identifying host responses that are diagnostic of disease severity; health authorities would not need a priori knowledge of an infectious agent to identify a disease outbreak. In this way, the BAS model proposed here extends syndromic monitoring approaches to describe the nature of identified diseases in greater detail and towards a more functionality-based perspective. Host signature-based approaches have previously been proposed for specific infectious organisms (e.g., Warsinske et al. [111]) and as a more generalized method for detecting multiple organisms [112,113]. Recent and continuing advances in signature discovery and multi-omics data analysis warrant further consideration of approaches that focus on BASs.

## Disclaimer

Pacific Northwest National Laboratory is a multiprogram national laboratory operated by Battelle Memorial Institute for the U.S. Department of Energy under Contract DE-AC06-76RLO. This manuscript was partially authored by Battelle National Biodefense Institute, LLC under Contract No. HSHQDC-15-C-00064 with the U.S. Department of Homeland Security. The United States Government retains and the publisher, by accepting the article for publication, acknowledges that the United States Government retains a non-exclusive, paid up, irrevocable, world-wide license to publish or reproduce the published form of this manuscript, or allow others to do so, for United States Government purposes. George Korch is the Laboratory Director, NBACC which is an FFRDC funded under Agreement No. HSHQDC-15-C-00064 which was awarded to Battelle National Biodefense Institute (BNBI) by the Department of Homeland Security (DHS) Science and Technology Directorate (S&T) for the management and operation of the National Biodefense Analysis and Countermeasures Center (NBACC), a Federally Funded Research and Development Center. No funds from this agreement were used for this effort. The views and conclusions contained in this document are those of the authors and should not be interpreted as necessarily representing the official policies, either expressed or implied, of DHS or the U.S. Government. The DHS does not endorse any products or commercial services mentioned in this presentation. In no event shall the DHS, BNBI or NBACC have any responsibility or liability for any use, misuse, inability to use, or reliance upon the information contained herein. In addition, no warranty of fitness for a particular purpose, merchantability, accuracy, or adequacy is provided regarding the contents of this document.
